# A Fast Alignment-Free Approach for De Novo Detection of Protein Conserved Regions

**DOI:** 10.1371/journal.pone.0161338

**Published:** 2016-08-23

**Authors:** Armen Abnousi, Shira L. Broschat, Ananth Kalyanaraman

**Affiliations:** 1 School of EECS, Washington State University, Pullman, WA, United States of America; 2 Paul G. Allen School for Global Animal Health, Washington State University, Pullman, WA, United States of America; 3 Department of Veterinary Microbiology and Pathology, Washington State University, Pullman, WA, United States of America; University of Michigan, UNITED STATES

## Abstract

**Background:**

Identifying conserved regions in protein sequences is a fundamental operation, occurring in numerous sequence-driven analysis pipelines. It is used as a way to decode domain-rich regions within proteins, to compute protein clusters, to annotate sequence function, and to compute evolutionary relationships among protein sequences. A number of approaches exist for identifying and characterizing protein families based on their domains, and because domains represent conserved portions of a protein sequence, the primary computation involved in protein family characterization is identification of such conserved regions. However, identifying conserved regions from large collections (millions) of protein sequences presents significant challenges.

**Methods:**

In this paper we present a new, alignment-free method for detecting conserved regions in protein sequences called NADDA (No-Alignment Domain Detection Algorithm). Our method exploits the abundance of exact matching short subsequences (*k*-mers) to quickly detect conserved regions, and the power of machine learning is used to improve the prediction accuracy of detection. We present a parallel implementation of NADDA using the MapReduce framework and show that our method is highly scalable.

**Results:**

We have compared NADDA with Pfam and InterPro databases. For known domains annotated by Pfam, accuracy is 83%, sensitivity 96%, and specificity 44%. For sequences with new domains not present in the training set an average accuracy of 63% is achieved when compared to Pfam. A boost in results in comparison with InterPro demonstrates the ability of NADDA to capture conserved regions beyond those present in Pfam. We have also compared NADDA with ADDA and MKDOM2, assuming Pfam as ground-truth. On average NADDA shows comparable accuracy, more balanced sensitivity and specificity, and being alignment-free, is significantly faster. Excluding the one-time cost of training, runtimes on a single processor were 49s, 10,566s, and 456s for NADDA, ADDA, and MKDOM2, respectively, for a data set comprised of approximately 2500 sequences.

## Introduction

Proteins play a vital role in living organisms. They are the main players in metabolic pathways, and to understand how cells work requires insight into the structure and knowledge of the function of a protein. A plethora of research to determine the structure and function of proteins has been conducted, but the rate of knowledge generated has grown much more slowly than the universe of identified proteins has. For example, between two releases of the UniProt Knowledgebase [1] (from release 2015_11 to 2015_12) only 287 sequences were added to the Swissprot section, which contains curated protein sequences with high levels of annotation. Over the same time period, the TrEMBL section (for automatically annotated sequences) has been augmented with more than 590,000 protein sequences. The need for more accurate and scalable automatic methods is compelling.

Variation in proteins comes from recombination and mutations in evolutionary modules [2]. These modules are generally known as *domains*. A single protein is made up of one or more domains. Thus, the detection of domains of protein sequences can be regarded as the initial step in domain family identification and protein clustering, which in turn can help in function and structure assignments. Accurate annotation of conserved regions, as building blocks of protein sequences, can also contribute to construction of evolutionary trees.

Many current approaches perform the detection of sequence domains by detecting only putative regions and as a part of domain family identification, i.e., protein clustering based on domains rather than as a separate question [3–5]. To the best of our knowledge there is no published research concentrating only on detecting conserved regions in proteins. Although correlation between the detection of domain regions and clustering based on domains appears to be natural, one can argue that a cluster is a global property of the universe of proteins, but a domain is a local property of each sequence which, as the definition suggests, needs to be common to many sequences. Global approaches to domain family identification also often involve the expensive operation of sequence alignment. Hence, fast and accurate detection of domain regions will contribute to the global operation of clustering proteins, both by improving the accuracy of detection and reducing runtime by removing expensive sequence alignment operations.

While the unprecedented growth in numbers of sequenced proteins has been a challenge for integration of automated methods in proteomics, the abundance of sequences also presents us with an opportunity: as the number of protein sequences containing domains increases, the frequency of occurrence of a particular domain also increases, making it easier to detect. Our task, then, is to extract high frequency regions from within the protein sequences. As we explain fully below, for each sequence, our alignment-free method translates its level of similarity to other sequences into a vector and then looks through each vector to detect the pattern within its indices. While we introduced the idea of this translation into a vector in [6], our experiments showed that our simple mathematical approach resulted in a heavy inter-dependence between the parameters selected for the method and the protein sequences. Here, we improve upon this previous work [6] by integrating a machine learning approach that reduces the dependence on parameters while improving the accuracy. We have also augmented our work with an extensive experimental results section in which we not only validate our proposed method but also provide comparison of our results with the results of other existing methods.

### Related Work

While detection of structural domains as opposed to functional domains has been an area of active research featured as a part of CASP experiments [7–9], the detection of sequence level conserved regions has been mostly overshadowed by efforts to cluster proteins based on their domain families [3–5]. In MKDOM2 [5], the shortest sequence (without repetitions) is marked as a domain and then PSI-BLAST [10] is used to detect regions of high similarity with that domain. Sequences containing regions similar to that domain are put in one cluster. The detected sections are removed from the sequences and the same operation is repeated to find other domains. The objective of MKDOM2 is expressed as identification of domain families rather than detection of domain regions themselves. MKDOM2 is an improvement over MKDOM [11] which in turn succeeded DOMAINER [12]. All three have been used to generate the ProDom protein family database [13]. Parallelization of MKDOM2 has been implemented using a master-worker structure [14].

In ADDA [3], an all vs. all BLAST [15] is performed on the entire data set. Based on the results of BLAST, a tree of putative domain regions is generated for each sequence. An optimization target is then used to find the final partitioning for each sequence. Unification is performed to construct a new graph based on final partitions and to cluster similar proteins together. In ADDA, domain boundary assessments are performed using the trees of putative domains. However, the final goal being domain family enumeration, unification is a crucial step and can possibly improve the partitions. An implementation of construction of ADDA putative domain trees of protein sequences from their all vs. all BLAST results has also been presented with support for parallel/multi-threaded executions.

In EVEREST [4] an all vs. all BLAST on the data set is followed by runs of the Smith-Waterman algorithm [16] on the selected sequences to generate a set of putative domain regions. After applying a series of different methods, including clustering the sequences based on detected putative domain regions, filtering low-scoring clusters using a boosting machine learning technique, and profile construction using ClustalW for each cluster, EVEREST finds a set of HMM profiles. The segments detected by these profiles replace the initial putative domain list and correspond to suggested domain families. This operation is repeated iteratively.

As can be seen, all three of these approaches focus on domain family identification rather than domain region detection, but they also use some methods for detection of putative domain regions as a preliminary operation for their final objective.

In this paper we present NADDA (No-Alignment Domain Detection Algorithm), an alignment-free, scalable method for detection of conserved regions in proteins. In the next section we present our approach to the problem. In the Results section we evaluate our method and compare its results with other methods. We conclude the paper by a brief discussion on what we achieved and prospective future work.

## Methods

Given a set *S* of *n* sequences, the problem of *conserved region detection* of protein sequences can be expressed as the demarcation of subsequences in each of the *n* sequences such that these subsequences are preserved over the evolutionary process. The preservation of these subsequences can be identified by a high local similarity score when the protein is aligned with other proteins of the same family. These conserved subsequences appear as motifs and sequence domains. Our method uses the frequency of short exact matching subsequences (*k*-mers) of a protein sequence in the data set as an indicator of the existence of a conserved region in the vicinity of that *k*-mer.

**Problem Definition:**

**Notation:** Given a sequence *s*, we denote the *i*^*th*^ character of *s* by *s*[*i*] and the substring of length *l* that starts at index *i* of *s* by *s*(*i*, *l*). We denote the length of a sequence *s* by |*s*|. A *k*-mer is a string of length *k*.

Additionally, let *S* = {*s*_1_, *s*_2_, …, *s*_*n*_} denote an input set of *n* protein sequences. We define:

**Definition 1** *k*-mer frequency: *The* frequency *of a k-mer in S is defined as the number of sequences in S that contain that k-mer at least once*.

A conserved window is then a substring of length *r*, centered at index *i*, where the average frequencies of *k*-mers in that substring is above a certain threshold *τ*. However, this definition is heavily dependent on the values of the threshold (*τ*) and the window size (*r*). To compensate for these limitations, we consider a smaller resolution of regions—indices. For each index *i* we consider a window of size 2 × *w* + 1 centered at *i*, and based on the frequency of *k*-mers inside this window we decide whether or not index *i* is part of a conserved region. The decision based on *k*-mer frequencies is not dependent on a single threshold *τ*; rather we find a set of thresholds {*τ*_1_, *τ*_2_, …, *τ*_2×*w*+1_} where *τ*_*j*_ is used as a threshold for the *j*^*th*^ *k*-mer in the window, i.e., the *k*-mer originating at index *i* − *w* + *j* − 1 of the sequence. This set of thresholds is found using our machine learning algorithm, and the prediction is also made by a combination of comparisons based on the thresholds.

**Definition 2** Conserved Region: *Given a sequence s ∈ S, a substring s(i, l) is said to be a* conserved region *if*:

1. *(conservation) every* *s*[*j*] *for* *j* ∈ [*i*, *i* + *l*] *is a conserved index, and*
2. *(maximality) neither the index* *s*[*i* − 1] *nor the index* *s*[*i* + *l* + 1] *(if either exists) is a conserved index*.

From the problem definition above, a simple approach is to compare each pair of sequences ($\left(\begin{array}{c} n\\ 2 \end{array}\right)$ pairs) and use the results to annotate the sequences with their conserved areas. To perform the alignment, one can use methods such as the Smith-Waterman local alignment [16] or related heuristics such as PSI-BLAST [10, 15]. However, these approaches are not scalable when |*S*| is large. Instead we generate a new representative vector for each sequence—*k*-mer profile as defined below—and perform a binary classification using feature vectors obtained from this representation. Rather than dissecting the sequence itself we dissect the profile vector into putative conserved regions and map these conserved regions onto the same indices of the sequence. As we will see in the Results section, the detected putative conserved regions widely correspond to the sequence domain regions of the sequences as they are annotated in well-known protein domain databases.

**Definition 3** *k*-mer profile: *The k*-mer profile *of a sequence s is a vector of the length of the sequence, where the value of each index is the frequency of the k-mer originating at that index in s*.

The problem of parsing a profile vector into putative conserved regions can be translated into a classification problem in which the task is to decide whether or not each index of the vector should be included in a conserved region. We generate an instance for each index of the profile vector and use a trained model to predict whether it belongs to a conserved region; this is a binary classification problem. Because the trained model depends only on the frequency values of each index in its profile vector rather than on the amino acid elements themselves, once we have a well-trained model it can be used for de novo detection of conserved regions. The details for each of these steps is explained below.

### Sequence *k*-mer Profile Generation

A *k*-mer profile is an alternative representation of a protein sequence. To construct a *k*-mer profile we need to count the number of times every *k*-mer appears in different sequences in our data set and record the counts at the same index at which the *k*-mer originates in the profile vector. We approach the problem using a two-pass hashing procedure. First, the algorithm reads every protein sequence and for each position index of the sequence computes the *k*-mer starting at that index. The computed *k*-mer is then used as a hash key to store the position index number and sequence ID for that *k*-mer in the hash table. If a *k*-mer appears multiple times in a sequence, we store all appearances as one entry in the hash table. Thus, the frequency of each *k*-mer in the data set will equal the number of entries stored for that *k*-mer in the hash table. When reading of the sequences has been completed, we enter the second pass of the algorithm. The algorithm uses the sequence IDs stored as hash values in the first hash table as the keys to construct a second hash table. The value of each sequence ID (hash key) in the second hash table is the originating index of each *k*-mer in that sequence along with the frequency of that *k*-mer. By ordering these frequencies in the hash table based on the index numbers, we essentially generate the *k*-mer profile for each sequence. The serial computation time for this step is *O*(*N*), where *N* is the total length of the *n* sequences in the data set. Because the operation on one sequence is independent of another, the algorithm is easily parallelizable. Later in this section we present a MapReduce [17] implementation.

### Classification Instance Generation

At the conclusion of the first step we have a set of *k*-mer profiles that are vectors with index values equal to the number of sequences in the data set that contain the *k*-mer initiating on the same index in the sequences. The next step is to generate classificaton instances for each index in each sequence of the *k*-mer profiles. Conversion of *k*-mer profiles to classification instances allows us to first train a binary classificaton model using known domain data and then to use the model to predict new domains. Given the *k*-mer profile *p* for sequence *s*, we generate one classification instance per index *i* in *s*. For such a profile and index, our classification instance is a vector *v* consisting of 2*w* + 1 elements (features) where the 2*w* + 1 elements are derived directly from the profile vector by copying the values from the window centered on index *i* of *p* and extending from both sides for *w* indices. These features represent the frequency of *k*-mers initiating at indices [*i* − *w*, *i* + *w*] of the corresponding protein sequence.

### Classification Model Construction

We train the classification model using classification instances that include a class label indicating whether or not the index associated with the instance is conserved. This is determined by querying domain databases. We also use these instances to test our model. Given the integer-valued features of our problem, the need to handle large data sets, and the non-linearity of the problem (due to the variable level of conservation among different domains), decision trees [18, 19] seem to be a natural choice to use for training the model. They support automatic feature selection and can learn different thresholds for different features. However, for small *w* the decision tree will not be able to see the overall picture of the *k*-mer profile and the model will lose robustness. The tree will be inclined to classify any short-lived increase in frequency as a domain index or a small local fall in frequency as a demonstration of absence of a conserved region. On the other hand, a larger *w* can result in overfitting the training data. To overcome the disadvantages of a simple decision tree classifier, we use a Random Subspace ensemble method [20] for which the base estimators are decision trees. This ensemble method constructs multiple decision trees on random subsets of features and classifies an instance based on the majority vote of the decision trees.

Because we are using *k*-mer profiles in our training set rather than actual protein amino acid sequences, our model depends only on frequencies (the values in the *k*-mer profiles) rather than on the amino acids. As a consequence, it can detect new conserved regions that were not present in our training set, given that there are enough sequences in the data set containing the conserved region to ensure the high frequency of *k*-mers in that region. In other words our algorithm is able to detect conserved regions that are present in multiple sequences in our data set, even if they were not annotated previously.

On the other hand, because the model is trained only once and we require accurate performance from that model when presented with new data, the selection of a good training set is crucial to the algorithm. First, the presence of conserved regions in too few sequences in the training set will result in small values in the *k*-mer profile and can be misleading to the training algorithm. Second, as we increase the size of the training set, we will be adding robustness to the trained classifier because conserved regions with varying frequency profiles will be represented in the training set. Using a large data set acquired from any of the big databases of protein conserved regions as our training set will satisfy both our conditions for the training set and will enable our algorithm to detect new conserved regions.

### MapReduce Algorithm for Profile Generation

We implement our algorithm in the MapReduce framework [17]. A MapReduce program consists of two main functions of *map* and *reduce*. In the map phase a set of mapper tasks generate KeyValues based on the input. These KeyValue objects are hashed based on their key and redistributed based on the result of the hash. The intermediate hash-based grouping phase is called the *shuffle* phase. Each reducer task then processes the set of values that are hashed to the same key. A reducer, in turn, can also generate KeyValue objects. The MapReduce paradigm suits data-parallel applications naturally. For our purpose, we exploit the shuffle phase to implement many of our hash-based functionalities in parallel.

In our MapReduce algorithm, initially each mapper reads the input set of sequences. It then generates KeyValue pairs using the *k*-mers of the sequences as keys and sequence ids and *k*-mer positions as values. The shuffle step subsequently sorts and redistributes these KeyValues such that each reducer receives one KeyMultiValue object, where all the values corresponding to the same key are sent to a single reducer. Based on the number of different sequence ids present in its MultiValue, each reducer determines the frequency of the *k*-mer mapped to it and emits a KeyValue, where the key is the sequence id and the value is the frequency of the *k*-mer and the position of the *k*-mer in that sequence. After this first complete stage of MapReduce, each reducer will contain frequencies of *k*-mers for each position in one protein sequence and can construct the *k*-mer profile for that sequence. The algorithm for this step is shown in Algorithm 1.

**Algorithm 1** Construction of *k*-mer profile (*S*:{*s*_1_, *s*_2_, …, *s*_*n*_}, *k*)

**for** each sequence *s*_*i*_ ∈ *S* with id $s_{id}^{i}$ **do**

 **for** each index *j* in *s*_*i*_ **do**

  *s*′ = *s*_*i*_(*j*, *k*)

  emit KeyValue $KV=<s^{\prime },\left(j,s_{id}^{i}\right)>$

 **end for**

**end for**

shuffle all KV’s

**for** each KeyMultiValue $KMV=<s^{\prime },\left(j,s_{id}^{i}\right),\left(m,s_{id}^{k}\right),\ldots ,\left(k,s_{id}^{l}\right)>$ **do**

 *count* = *number* *of* *different* *s*_*id*_ *in* *the* *KMV*

  **for** each value $V=(j,s_{id}^{p})$ in *KMV* **do**

  emit KeyValue $<s_{id}^{p},\left(j,count\right)>$

 **end for**

**end for**

shuffle all KV’s

**for** each $KMV=<s_{id}^{i},\left(j_{1},count_{j1}\right),\left(j_{2},count_{j2}\right),\ldots >$ **do**

 sort values based on *j*’s

 return the *count*s based on sorted *j*’s

**end for**

### Implementation and Software Availability

We have implemented our method, NADDA, in C++ and Python. The C++ code uses the *MRMPI* library [21] for MapReduce and the *BOOST* library. The Python code uses the *scikit-learn* (v. 0.17) machine learning library [22]. Software is available as open source at https://bitbucket.org/armenabnousi/nadda.

## Results

### Experimental Setup

Eleven different data sets were used for our experiments, one consisting of approximately 50,000 bacterial protein sequences and the remainder consisting of smaller sets of a few thousand protein sequences each. Some of the latter sets consist entirely of bacterial protein sequences; others are mixtures of sequences from bacteria, eukaryota, and archaea. The number of sequences and percentage of bacterial sequences in each of the smaller sets are listed in Table 1. More information regarding the domains in each of these is shown in Tables 2 to 11. The smaller sets (#1-#10) were used to evaluate *de novo* detection of conserved regions with our model and to compare it with MKDOM2 [5], ADDA [3], and InterPro [23]. The large data set #11 (Table 12) was used to train and test our classification model. Because both MKDOM2 and ADDA require sequence alignment, an expensive operation, it was infeasible for us to conduct a comparison using our largest data set. For example, MKDOM2 had not completed its run on data set #11 after 20 days. It should also be noted that both methods focus on clustering proteins based on domain families rather than on detection of conserved regions. MKDOM2 clusters are generated based on regions in sequences with high sequence similarity. These regions represent conserved regions as defined earlier. Similarly, as an initial step ADDA selects some putative domain regions. An optimization objective is then used to generate final domain boundaries on each sequence. These boundaries are later used to construct a graph and perform clustering. While each identified region (indices enclosed between two consecutive boundaries) is expected to contain exactly one domain, because they are used as an intermediate result ADDA does not guarantee that a complete domain will be captured in each domain region predicted by the optimization procedure (i.e., it does not guarantee that there will not be any non-domain indices inside the predicted region). However, a comparison can still be made.

**Table 1:** Structure of small data sets (#1-#10) used for evaluation of de novo detection of conserved regions and for runtime studies.

Data Set	#sequences	% bacteria	% archaea	% eukaryota
#1	1,424	100%	0%	0%
#2	1,542	100%	0%	0%
#3	1,479	100%	0%	0%
#4	1,494	100%	0%	0%
#5	2,037	95.4%	2.6%	2.0%
#6	808	93.1%	3.4%	3.5%
#7	2,565	63.4%	1.2%	35.4%
#8	2,031	48.9%	0	51.1%
#9	2,138	29.5%	1.7%	68.8%
#10	1,938	11.4%	1.8%	86.8%

**Table 2:** Data Set #1.

Domain Name	#sequences
FAD_binding_9	750
FixS	350
Gas_vesicle	368
total	1,424

**Table 3:** Data Set #2.

Domain Name	#sequences
Caa3_CtaG	499
Dak1_2	699
dCache_3	351
total	1,542

**Table 4:** Data Set #3.

Domain Name	#sequences
XisI	213
NapB	179
EutN_CcmL	330
LptC	823
total	1,479

**Table 5:** Data Set #4.

Domain Name	#sequences
EpsG	313
RcnB	207
FlgN	542
Lipoprotein_17	353
LolA_like	344
total	1,494

**Table 6:** Data Set #5.

Domain Name	#sequences
NA37	384
DbpA	1,232
AAA_PrkA	426
total	2,037

**Table 7:** Data Set #6.

Domain Name	#sequences
NA37	384
AAA_PrkA	426
total	808

**Table 8:** Data Set #7.

Domain Name	#sequences
Rad4	693
YccF	1,034
DbpA	1,232
total	2,565

**Table 9:** Data Set #8.

Domain Name	#sequences
RbsD_FucU	567
Vasohibin	180
NA37	384
Ndc1_Nup	431
total	2,031

**Table 10:** Data Set #9.

Domain Name	#sequences
AAA_PrkA	426
Dnal_N	140
FTCD	262
FACT-Spt16_Nlob	433
SAD_SRA	890
total	2,138

**Table 11:** Data Set #10.

Domain Name	#sequences
Has-barrel	135
EccE	122
EFhand_Ca_insen	724
KA1	959
total	1,938

**Table 12:** Common bacterial protein domains used for construction of data set #11.

Protein Domain Name	Number of Sequences
TOP1Bc	11,545
CBM_2	726
ZnMc	2,967
ZipA_C	1,508
HLH	16
NADH-G_4Fe-4S_3	5,476
POLAc	7,110
PP2Ac	907
Resolvase	16,007
S_TKc	1,519
Endonuclease_NS	2,435
total	50,214

To obtain our large data set (#11), we queried the SMART [24] protein domain database (http://smart.embl-heidelberg.de) using eleven common bacterial protein domains listed in Table 12. This resulted in a list of 50,214 bacterial protein sequences that included one or more of the eleven domains. We then queried Pfam-A [25] release 29.0 with each of these sequences and labeled the domain indices to use as ground-truth for our training instances. For the smaller mixed protein data sets, we randomly selected some protein families through the Pfam webpage (http://pfam.xfam.org; v.29) and then ran Pfam 29.0 on those sequences to mark their Pfam domain regions. Similarly, for the predominantly bacterial protein data sets, we either randomly identified bacterial protein families through the Pfam webpage or else used domains from the other data sets that had a high bacterial percentage. Again, we marked their domain regions using Pfam 29.0. Tables 2 to 11 show the protein families selected from the Pfam online database and the number of sequences present in each data set. Totals in these tables are computed after removing common redundant sequences. We then queried InterPro (v5.18-57) using each of our 10 smaller data sets.

Comparisons were performed based on the coverage of the domain indices reported by Pfam-A in our findings and in the MKDOM2 and ADDA results as well as on the coverage of conserved region indices reported by InterPro in the NADDA results. We measured coverage using the three values of accuracy (AC), specificity (SP), and sensitivity (SN) of the detected conserved indices as defined respectively by:

$$AC=\frac{\left|TP|+|TN\right|}{N}$$

$$SN=\frac{\left|TP\right|}{\left|TP|+|FN\right|}$$

$$SP=\frac{\left|TN\right|}{\left|TN|+|FP\right|}$$

where *TP*, true positives, indicate the indices in the data set that are marked as part of a domain region in Pfam-A, and the detection method also has classified them as conserved indices; *TN*, true negatives, are the indices that both Pfam-A and the conserved region detection method have not included in any domain/conserved region; *FP*, false positives, are the indices that are not marked by Pfam-A as contained in a domain, but the detection method has classified them as conserved indices; and *FN*, false negatives, are the indices that are included in a Pfam-A domain, but the detection method has fallen short of identifying them as conserved. Finally, *N* is the total length of the sequences in the set (*N* = |*TP*| + |*TN*| + |*FP*| + |*FN*|).

For our experiments, unless otherwise stated, values for *w* (where 2*w* + 1 is the size of the feature vector), *k* (size of each *k*-mer), *MSS* (which indicates the level of pruning of the decision tree—minimum number of instances in each internal node), and *max*_*features* (number of features used in each decision tree for the ensemble method) are set respectively to 10, 6, 100, and 7. We discuss parameter selection in greater detail in the Supporting Information (S1 File).

### Evaluation of Results

We evaluate our results in three phases. First, we evaluate our training method by partitioning data set #11 into training and test sets. This is the common practice in the machine learning community for evaluation of a training method. The second phase includes comprehensive experiments evaluating overall performance of NADDA by testing it using data sets with domains that are new to NADDA (data sets #1—#10). Here we also compare NADDA with ADDA and MKDOM2. Finally, in the third phase, we compare NADDA against InterPro, re-evaluating its ability to capture regions that are missed in Pfam. In addition, we provide some example sequences from data sets #1-#10, comparing our detected conserved regions with the segments annotated by InterPro.

**Evaluating the training method:** In the first phase of our experiments we perform two different types of training-set/test-set partitioning of data set #11, training the model on a training set and measuring the results on a test set. First, we divide data set #11 by randomly selecting 20% of the sequences to be included in the test set and the rest in the training set. The second partitioning is performed by selecting 20% of the domains from data set #11 that are present in more than 50 sequences and including every sequence that contains one (or more) of these selected domains in the test set. The reason for setting a threshold for presence of a domain in a sequence in order to be considered a candidate for the test set is explained in the Supporting Information section(S2 File). We refer to the first train/test partition as repetitive and the second one as non-repetitive. The reason for the two different partitions is that while our repetitive partitioning method is widely accepted in the machine learning community, here it can result in overfitting. Because our method depends on the frequency of repeating *k*-mers, we require that multiple sequences possibly very similar to each other be present in our data set. Dividing the data based on random selection of sequences might result in having similar sequences in the training and test sets. The results achieved might then represent the case when the domain in the query sequence has already been discovered in similar sequences and we are only trying to retrieve this knowledge. In contrast, for our non-repetitive partitioning, because we withhold a set of domains from the training set, the results are better representative of discovering new domains that were not previously known as well as domains that were previously known. This partition is better representative of the results we would obtain using the current knowledge-base of protein conserved regions for new sequences.

The results for the two sets are shown in Table 13. For the repetitive case the accuracy is 83% with 96% recall (sensitivity). For the non-repetitive case, as expected, the numbers decrease. However, we can still predict with 80% accuracy whether or not a location in a protein sequence is included in a conserved region.

**Table 13:** Performance of NADDA based on Pfam; When similar domains are present in the training set (repetitive) and when some domains are withheld from the training set (non-repetitive).

	AC	SN	SP
Repetitive	83.4%	96.9%	44.1%
Non-repetitive	80.5%	95.7%	25.3%

**Comparison with other methods against Pfam:** In the second phase of the evaluation we compare our results with final sequence segments produced by MKDOM2 in clustering based on domain families and with final partitions found by the optimization method used in ADDA assuming that Pfam domains represent the ground-truth. As mentioned earlier, MKDOM2 performs clustering by selecting the shortest possible subsequence to be considered as a conserved region and aligning that subsequence with the remaining sequences. The final reported subsequences that have been aligned with each other represent conserved regions on the sequences and can be compared to our detected conserved regions. Similarly, ADDA performs clustering by first selecting a set of putative domain regions and optimizing the boundaries between these provisional domains to construct a protein similarity graph based on detected domains. Although clustering is performed after graph construction and additional filters in these later steps can affect the quality of ADDA clustering, we can use the optimized domain boundaries to compare their pre-computed domains with our method.

**Fig 1:**
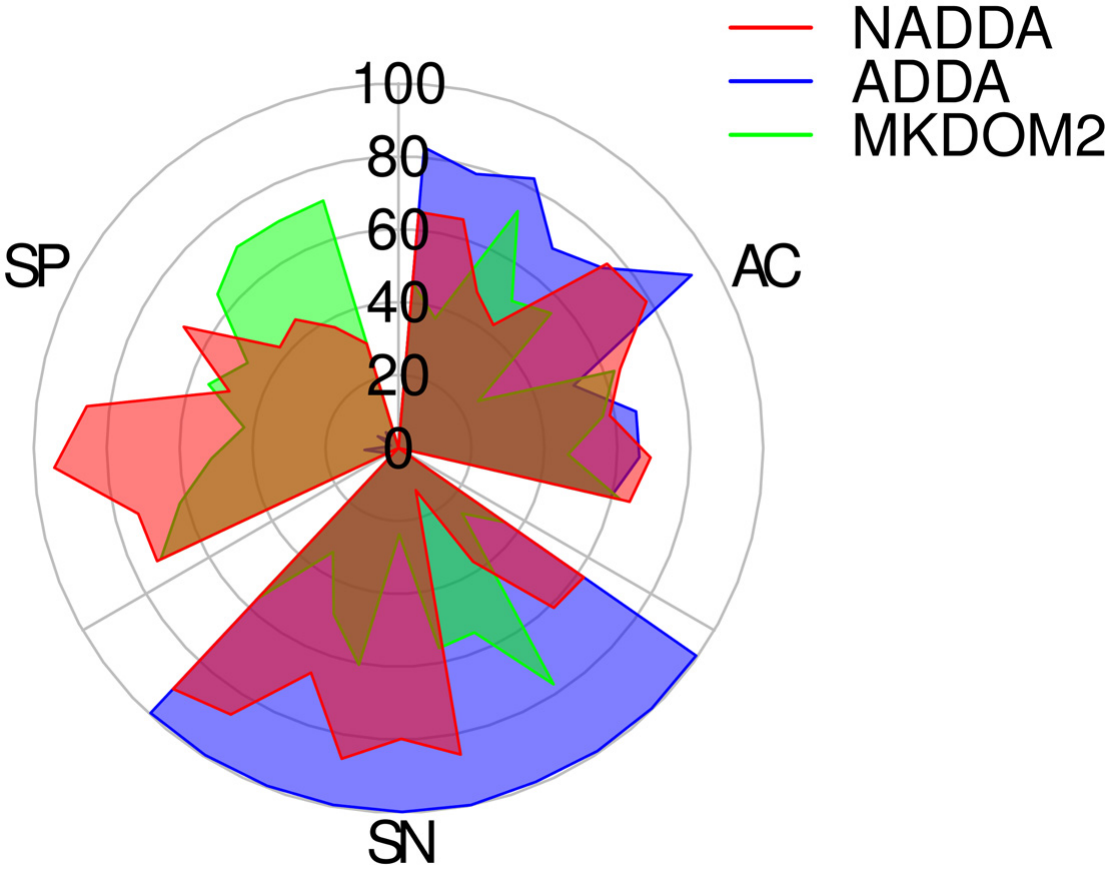
Comparison of results from NADDA, ADDA, and MKDOM2 with Pfam on small data sets. (AC = accuracy, SN = sensitivity, SP = specificity).

We trained our model using data set #11 and tested using data sets #1-#10. Because other methods depend on pairwise sequence similarity and BLAST results, we were forced to pick smaller sets for testing purposes. As mentioned earlier, MKDOM2 ran for 20 days on data set #11 without completion, and ADDA took long even for the small data sets as we will show later in Runtime Study section. We used MKDOM2 from the Xdom2.0 [5] package and the ADDA implementation obtained from its webpage (using the default settings: *K* = 73.70676, *C* = 8.33957, *E* = 0.05273, *M* = 1.417, *N* = 0.008 and *r* = 1, except for 100 maximum iterations (*i* = 100); in many of the cases the program stopped after only a few iterations. We also used BLAST+ v2.2.31 to generate the input files for ADDA). Fig 1 represents our findings. The circle displays our three metrics of accuracy (AC), sensitivity (SN), and specificity (SP) for the three different methods: ADDA (blue), MKDOM2 (green), and NADDA (red). The outermost circle represents 100% of the related measure and the center of the circle represents 0%. For each section (AC, SN, and SP) the bacterial percentage of the protein sequences in the data set for each point decreases in the clockwise direction; the first four points in each section correspond to 100% bacterial data sets. Table 14 shows the detailed results for the same measurements. NADDA shows higher accuracy for all but two of the test sets when compared to MKDOM2 and higher or comparable accuracy for many of the sets when compared to ADDA. MKDOM2 generally has better specificity scores (average 60.7%) than both ADDA and NADDA while its sensitivity scores are worse (average 51.5%). The advantage of specificity over sensitivity in MKDOM2 may be a result of two factors. First, MKDOM2 uses a high-cut off value in PSI-BLAST for the sake of runtime during its heuristic sequence alignment, thereby trading off sensitivity. Second, the greedy nature of the MKDOM2 algorithm inhibits precise detection of domain regions. ADDA shows higher than 99% sensitivity in all cases but very low specificity (4.6% on average). This is because the optimization method in ADDA tries to detect putative domain boundaries rather than domains themselves and the results are later used for graph construction together with some filters in order to achieve final clustering. As a result, ADDA does not care about mislabeling extra positions as long as the boundary between two domains is preserved. These extra indices could possibly be filtered in the graph construction and clustering process.

**Table 14:** Performance of the algorithm compared to other methods based on Pfam.

Data Set	Percentage Bacterial	Mean Freq.	Freq. Variance	NADDA			ADDA			MKDOM2		
				AC	SN	SP	AC	SN	SP	AC	SN	SP
#1	100%	5.9	169.1	64.9%	61.9%	73.0%	82.7%	99.5%	3.7%	45.4%	35.5%	72.0%
#2	100%	13.4	3,386.4	65.1%	61.1%	73.5%	78.1%	99.6%	4.9%	36.9%	25.0%	61.8%
#3	100%	2.5	30.2	47.8%	37.2%	94.5%	82.7%	99.4%	9.3%	72.7%	77.5%	51.4%
#4	100%	1.4	3.3	42.6%	12.5%	86.2%	69.1%	99.0%	3.1%	50.9%	54.7%	42.7%
#5	95.4%	36.3	13,466.3	76.3%	85.7%	48.9%	74.6%	99.9%	2.2%	55.8%	56.0%	54.9%
#6	93.1%	23.7	1,833.9	78.9%	79.7%	67.6%	93.3%	99.8%	6.5%	25.4%	23.7%	47.5%
#7	63.4%	25.2	9,628.8	64.5%	86.6%	42.6%	51.0%	99.5%	3.3%	62.8%	60.4%	65.1%
#8	48.9%	10.8	522.6	58.6%	66.1%	45.1%	65.9%	99.4%	5.5%	56.6%	48.9%	70.7%
#9	29.5%	18.2	1,108.6	69.1%	86.3%	37.4%	66.1%	99.5%	4.2%	46.5%	33.7%	70.3%
#10	11.4%	54.8	7,183.5	65.0%	90.4%	29.9%	59.7%	99.5%	3.5%	62.5%	56.5%	70.9%
Average	74.17%	19.22	3,733.26	63.3%	66.7%	59.9%	72.3%	99.5%	4.6%	51.5%	47.1%	60.7%

In comparison to the other two methods, we can see that for 4 out of 10 test sets, NADDA exhibits higher than 80% sensitivity. The sensitivity is significantly low for data sets #3 and #4. It is noticeable that for these sets the mean and variance for the *k*-mer frequency are also smaller than for the other sets. In general there is a correlation between the accuracy of our method and the variance of the *k*-mer frequency. In fact, the frequency variance depends on our choice of *k*, and it will increase as *k* is decreased. However, if *k* is too small, the variance will be high due to random exact matches that do not signify real conservation. This is discussed further in the Supporting Information section (S3 File).

**Fig 2:**
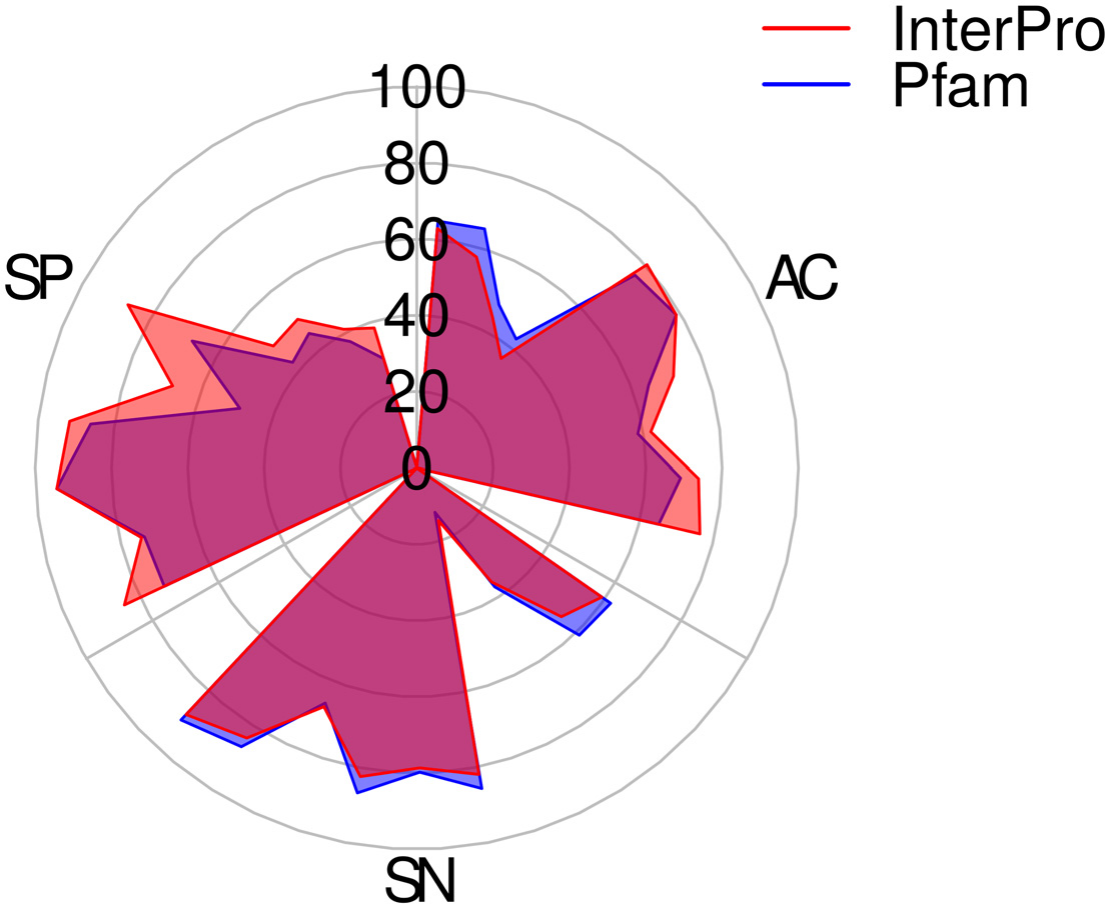
Comparison of NADDA with InterPro and Pfam using small data sets. (AC = accuracy, SN = sensitivity, SP = specificity).

**Comparison against InterPro:** In the last phase of our evaluation, we compare conserved indices detected by NADDA with regions annotated by InterPro [23]. InterPro uses predictive models (signatures) generated from multiple databases, including Pfam, SMART, ProDom [13], prosite [26], TIGRFAMs [27], etc. InterPro annotates domains as well as motifs (short conserved regions). As such, there should be more similarity between NADDA and InterPro. We compared our results with InterPro regions. The results are shown in Fig 2. Because InterPro includes Pfam, replacing our Pfam annotations with InterPro annotations will only add to the indices marked as conserved in our data sets. This can be manifested by a decrease in our |*FP*| and |*TN*|. Indices that we had marked as conserved but Pfam had not might be detected as conserved in InterPro as well, resulting in a smaller |*FP*|. Similarly indices that neither NADDA nor Pfam detected as conserved might be marked as conserved in InterPro, resulting in a decrease in |*TN*|. A decrease in |*FP*| will result in improved *SP*.

It should also be noted that for this comparison we are still using the model trained using Pfam annotations for data set #11. Although this can add to the false examples in the training set, the results show an increase in *SP* as expected, indicating the robustness of our training method.

**Fig 3:**
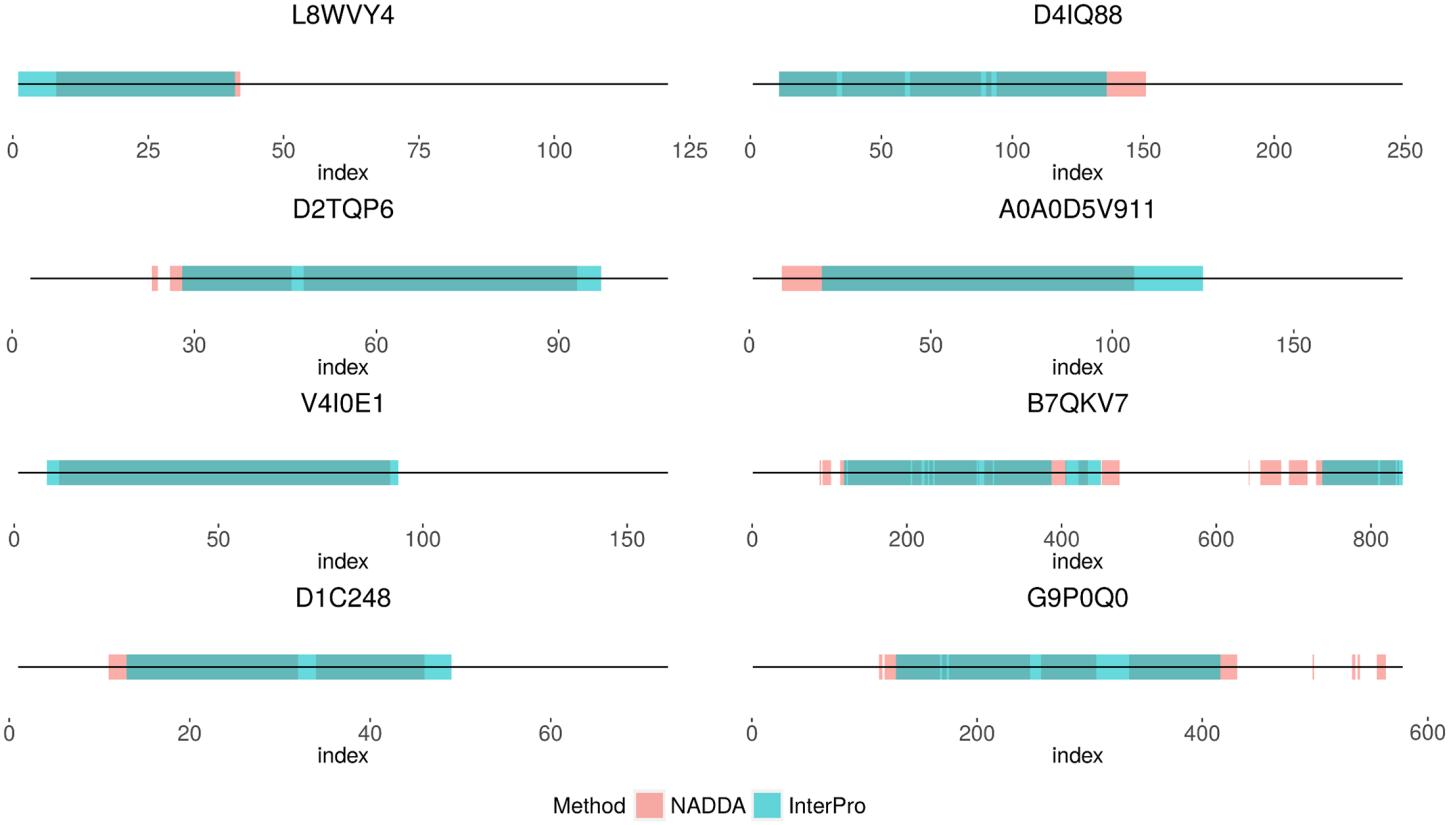
Comparison between the NADDA outputs and InterPro annotations for a few example sequences.

Fig 3 shows some example results from our experiments. Training is performed using data set #11, while the sequences shown are from the smaller data sets (#1-#10). The domains present in the smaller data sets are not present in the training set. These examples demonstrate that in many of the indices, the output from NADDA matches with the InterPro annotated regions.

### Parametric Study

The parameters used in our method are *k*, the size of a *k*-mer; *w*, where 2*w* + 1 is the number of features used in a classification instance; *MSS*, indicates the level of pruning of the decision tree; and *maximum*_*features*, number of features used in the ensemble method. The parameters *w*, *MSS*, and *maximum*_*features* were set to avoid overfitting and underfitting as explained in the Supporting Information (S1 File). The optimal value for *k* was found empirically from the set {3, 4, 5, 6, 7, 8}. We have shown the effect of varying *k* in the Supporting Information (S3 File). Varying *k* affects the mean frequency of *k*-mers as well as their variance and, thus, is important in the performance of the method.

### Runtime Study

We performed a runtime study by comparing the time for a serial run of NADDA with that of ADDA and MKDOM2 and also by a scalability evaluation of NADDA in a parallel environment.

We ran the algorithms on our in-house Linux cluster which includes 8 nodes of 64 AMD processors (2.29GHz), each node having 128GB shared memory. We used ADDA and MKDOM2 implementations as described earlier.

Table 15 lists a comparison of runtimes for NADDA, ADDA, and MKDOM2 for data set #7 on a single processor. The runtime for our method does not include the one-time training time. The ADDA runtime presented in Table 15 includes the time required for running BLAST.

**Table 15:** Runtimes of different methods for Data Set #7. The times represented here are for a serial run, i.e., single core. As shown in Fig 4, NADDA scales almost linearly in the number of cores, allowing the use of the method on even larger data sets.

Method	Time (s)
NADDA	49
ADDA	10,566
MKDOM2	456

**Fig 4:**
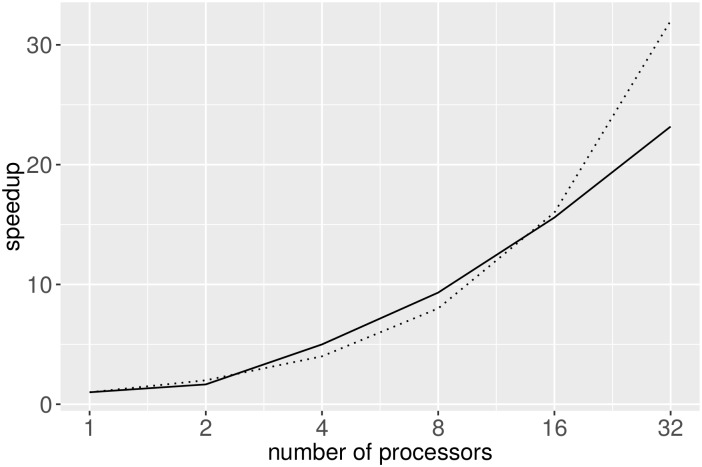
Speedup for parallel execution of NADDA on data set #11. The dotted line shows the ideal linear speedup; the solid line is the actual speedup using NADDA.

We can see that even for a small set of about 2,500 sequences, NADDA finishes much more quickly than the other methods. We ran the parallel implementation of our code on data set #11 (50,000 sequences) and showed that it scales nearly linearly with an increasing number of processors (Fig 4). NADDA took 79 seconds using 32 processors to complete this data set given an already trained model. Training was not parallelized and on data set #1 took 61 minutes. Parallel constructions for decision trees are proposed [28–30]. A detailed study of our parallel runtime is presented in the Supporting Information section S4 File.

## Conclusions and Future Work

We presented the problem of detection of conserved regions in protein sequences. In the past, multiple methods have been proposed for detection of putative domain regions from protein sequences. However, to the best of our knowledge there is no prior work that focuses on the detection of conserved regions, given a large collection of protein sequences. The method proposed in this paper fills this gap using an alignment-free approach.

We showed that rather than using an amino acid sequence of a protein, we can utilize its vectorized representation for our computation. We presented *k*-mer profiles of proteins as a new representation for protein sequences which can be useful for increasing the scalability of computation. We presented a MapReduce algorithm for generation of *k*-mer profiles. We used a random subspace ensemble learning method to improve the accuracy of conserved region detection.

Our experiments show competitive accuracy, sensitivity, and specificity measures for our method when compared to other methods. We also showed that our parallel implementation is scalable and works on large data sets. Our experiments show near-linear speedup.

We showed that our method is able to detect conserved regions of bacterial protein sequences as well as conserved regions of eukaryota and archaea protein sequences. The category of the species does not affect the results of our method. Moreover, we showed that variance of the *k*-mers from their mean has a correlation with the ability of our method to detect conserved regions. When the variance is low, we may be able to decrease *k* to obtain higher variance and consequently higher accuracy. Extremely small values of *k*, however, can result in higher variance due to random exact matches and should be avoided.

Use of structured prediction techniques might increase the accuracy of our algorithm. However, a structured prediction model will likely give higher weights to the context (previous predictions) rather than to other features. Another problem with use of different machine learning algorithms is the large scale of our data.

The iterative MapReduce algorithm fits best in the Spark parallel computing model [31] which also provides machine learning tools. In future work, we will implement our algorithm using the Spark paradigm.

## Supporting Information

S1 File Parameter Selection. (PDF)

S2 File Partitioning the Data Set. (PDF)

S3 File Varying the *k*-mer Size. (PDF)

S4 File Parallel Performance. (PDF)
