# Supplementary material for: A Fast Alignment-Free Approach for De Novo Detection of Protein Conserved Regions
Source: PLoS One. 2016 Aug 23;11(8):e0161338. doi: 10.1371/journal.pone.0161338 (PMC4995020; doi:10.1371/journal.pone.0161338)
Supplement: S1 File — (PDF) [file pone.0161338.s001.pdf]

S1 Parameter Selection

NADDA uses the frequency of k-mers to detect conserved regions. The presence of multiple sequences including similar conserved regions is a prerequisite for high frequencies and a greater probability for correct classification. Generally, we do not want to remove redundant highly-similar sequences from our data set. However, to select the correct parameter settings, we have to remove the dependence of the parameters on the redundant sequences in the data set which can otherwise result in overfitting. To overcome this problem, we used CD-HIT [1, 2] to extract a subset of sequences with less than 40% sequence identity from data set #11. This subset is composed of 3,694 sequences. Then we used the original data set (#11) to compute the frequencies but used only the 3,694 sequences of the subset to select our parameters.

We experimented with various values of w (where $2w + 1$ is the number of features) from $\{5, 10, 20, 30, 40, 50, 60, 70\}$ and ran cross-validation with different values for MSS (where MSS is used to prune our decision tree) and for $max\_features$ (the number of features used in each decision tree). As we increased the value of w, the algorithm started to overfit, ignoring the effect of extraction of sequences with lower sequence identity. The cross-validation tended to select higher values for $max\_features$ while improving the accuracy by less than a percent. Consequently we selected $w = 10$ as the number of indices before and after each index to be included in the feature vector of that index.

We ran ten-fold cross-validation using values from $MSS = \{50, 60, 70, 80, 90, 100, 110, 120, 130, 140\}$ and $max\_features = \{5, 6, 7, 8, 9, 10, 11, 12, 13, 14\}$, and based on the results of this procedure we set $MSS = 100$ and $max\_features = 7$. The selection of k was performed based on observations described in the Parametric Study section.

References

1. Li W, Godzik A. Cd-hit: a fast program for clustering and comparing large sets of protein or nucleotide sequences. Bioinformatics. 2006;22(13):1658–1659.

2. Fu L, Niu B, Zhu Z, Wu S, Li W. CD-HIT: accelerated for clustering the next-generation sequencing data. Bioinformatics. 2012;28(23):3150–3152.
