# Supplementary material for: A Fast Alignment-Free Approach for De Novo Detection of Protein Conserved Regions
Source: PLoS One. 2016 Aug 23;11(8):e0161338. doi: 10.1371/journal.pone.0161338 (PMC4995020; doi:10.1371/journal.pone.0161338)
Supplement: S2 File — (PDF) [file pone.0161338.s002.pdf]

Figure A. Distribution of number of sequences containing each domain in data set #11

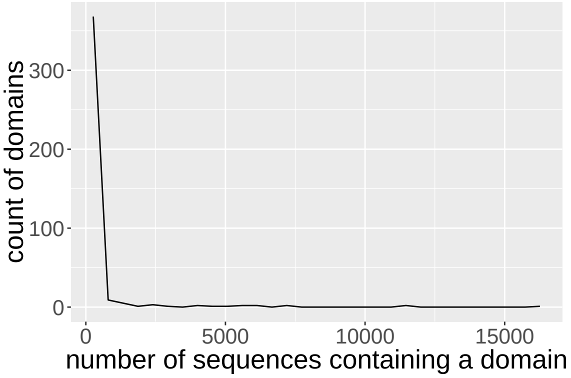

S2 Partitioning the Data Set

We partitioned data set #11 in two different ways. First, we randomly selected 20% of the sequences to construct the test set and used the remaining 80% for the training set. We call this division a repetitive partition because all the conserved regions we want to detect from the test set that are repeated in many other sequences are possibly already present in our training set via other sequences sharing the domains. In the second method, which we call an non-repetitive partition, we selected 20% of the domains. Any sequence that contains one or more of the selected domains is put into the test set. However, our selection of 20% of the domains is not from the complete pool of domains present in the data set, rather we consider a lower limit (50) on the number of sequences that contain a domain in order to take that domain into consideration. The reason for selection of domains with more than 50 sequences is two-fold: The distribution of the number of sequences containing each domain from data set #11 is shown in Fig. A. We see that many of the domains are present only in a few sequences. If we select any 20% of the domains without considering such a threshold, we are highly likely to end up having a very small test set, hindering an effective evaluation of the method. Second, without a lower limit on the number of sequences containing a domain, many of the domains randomly selected for the test set might be under-represented in the data set. Our algorithm or any other method that is based only on amino acid sequences clearly cannot detect such under-represented domains.
