# Supplementary material for: A Fast Alignment-Free Approach for De Novo Detection of Protein Conserved Regions
Source: PLoS One. 2016 Aug 23;11(8):e0161338. doi: 10.1371/journal.pone.0161338 (PMC4995020; doi:10.1371/journal.pone.0161338)
Supplement: S3 File — (PDF) [file pone.0161338.s003.pdf]

Figure B. Statistics for classification results on data set #9 using varying values of k (k-mer size)

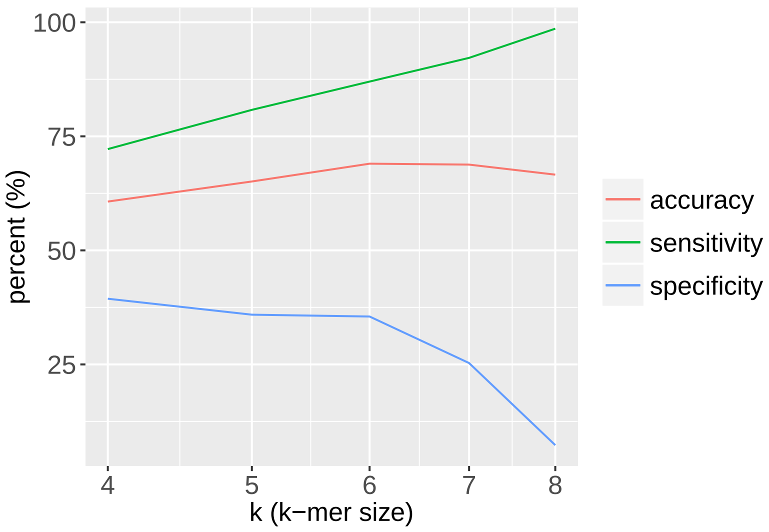

S3 Varying the k-mer Size

In selecting k there were not many viable choices. As we increased k, many k-mers were contained in a conserved region according to Pfam, but they were not repeated in the data set. More specifically for $k = 8$, there were many sequences from data set #11 for which the k-mer profile was a vector of 1's. Such a k-mer profile does not present any useful data for our machine learning algorithm. The training algorithm learns that even small values in features can represent conserved regions and the trained model will be biased. As we decreased k to 4, we observed increasing variance in values in each k-mer profile. For $k < 4$ we observed random repetitions of k-mers in sequences, and therefore we could not conclude that a high number of repetitions was due to conservation.
Fig. B shows the accuracy of prediction on set #9 for different values of k using a model trained on set #11. Practical values are $4 \leq k \leq 7$; we used $k = 6$ which in average gives the best results. Other parameters were set to $w = 10$, $MSS = 100$, and $max\_features = 7$. As we incremented k, specificity decreased while sensitivity increased, indicating the tendency of the algorithm to classify more indices as conserved, resulting in higher number of true positive predictions and thus higher sensitivity but lower specificity.

We noted the correlation between variance from the mean for k-mer frequencies and prediction accuracy in our results section. As shown in Table 5 in the main paper, if the variance is too low, the method is unable to make correct predictions. Low variance data does not give much information about the sequences: if the mean is high (compared to the mean frequency in the training set), the method will predict most of the indices as conserved and if the mean is low, most of the indices will be marked as non-conserved. On the other hand selecting a smaller k will result in higher variance; however, if the variance is high due to very small k, that variance might be a result of random exact matches which is misleading. The accuracy for data set #9 using smaller k-mer sizes does not deteriorate significantly. This is due to the higher variance of k-mer frequencies even for smaller k's. For data set #3, the smaller k-mer sizes results in higher variance but also a drastic decrease in accuracy. This is shown in Table A.

Table A. Correlation between variance and accuracy

		4-mer	5-mer	6-mer	7-mer	8-mer
Data set #9	Accuracy	60.3%	65.0%	69.0%	68.8%	66.8%
	Variance	3357.19	1672.57	1108.69	839.96	672.35
	Mean	49.1	24.1	18.2	15.0	12.8
Data set #3	Accuracy	26.0%	41.9%	49.1%	-	-
	Variance	190.67	64.52	30.2	-	-
	Mean	9.1	3.7	2.5	-	-
