# Supplementary material for: A Fast Alignment-Free Approach for De Novo Detection of Protein Conserved Regions
Source: PLoS One. 2016 Aug 23;11(8):e0161338. doi: 10.1371/journal.pone.0161338 (PMC4995020; doi:10.1371/journal.pone.0161338)

S4 Parallel Performance

The runtime for MKDOM2 increases exponentially with the size of the input sequences [1]. In [1], it is shown that a parallel implementation of MKDOM2 takes more than an hour for an input file of 21.5MB using 8 processors, while the runtime for NADDA using 8 processors and for data set #11 (33MB) is about three minutes. ADDA also provides parallelization in generating putative domain trees for sequences that are later used as input for the ADDA optimization step. For one processor tree generation is the operation that contributes the most to the ADDA runtime. If we use parallel tree generation, the initial all vs. all BLAST is the most expensive operation for ADDA; for data set #7 all vs. all BLAST took about 58 minutes. For larger data sets, NADDA, which is completely parallelizable, is incomparable in runtime to the other two methods.

We examined the scalability of our method by running it on a different number of processors on our Linux cluster. We used our MapReduce implementation of k-mer profile generation using the MRMPI library [2][1]. Given a trained model, decision making for each query sequence depends only on its own k-mer profile and the model, which enables us to run the model on multiple query sequences in parallel. After generation of k-mer profiles, we loaded the profiles and a model trained using *scikit-learn* machine learning library [3] for Python. We then queried the profiles on the model. We used *mpi4py* library [4] to run the queries in parallel.

Fig. 4 (in the main paper) showed the speedup of NADDA using a varying number of processors on data set #11 ($\tilde{5}$0,000 sequences). The dotted line illustrates the desired linear speedup. We can see that for up to 16 processors, the speedup of our method is near linear. For 32 processors the speedup reduces which should be due to the small size of the data set. As we increase the size of the data set, we will see better speedup for a larger number of processors.

Fig. C shows the runtime breakdown for different stages of NADDA when run on a different number of processors. In the current implementation the profiles are written down to disk after the profile generation step and then read back through the Python script in the instance construction step. In Fig. C we see that the profile generation step stops scaling for more than 8 processors. This is due to the small size of the input data and the disk output at the end of this stage. This same disk I/O can be the contributing factor to the super-linear scalability for the smaller number of processors.

The training time using a single processor for data set #11 as the training set is 61 minutes.

References

1. Kahn D, Rezvoy C, Vivien F. Parallel large scale inference of protein domain families. In: Parallel and Distributed Systems, 2008. ICPADS'08. 14th IEEE International Conference on. IEEE; 2008. p. 72–79.

2. Plimpton SJ, Devine KD. MapReduce in MPI for large-scale graph algorithms. Parallel Computing. 2011;37(9):610–632.

3. Pedregosa F, Varoquaux G, Gramfort A, Michel V, Thirion B, Grisel O, et al. Scikit-learn: Machine Learning in Python. Journal of Machine Learning Research. 2011;12:2825–2830.

4. Dalcin LD, Paz RR, Kler PA, Cosimo A. Parallel distributed computing using python. Advances in Water Resources. 2011;34(9):1124–1139.

[1]http://mapreduce.sandia.gov

Figure C. Runtime breakdown of NADDA for parallel execution of model on a varying number of processors on data set #11

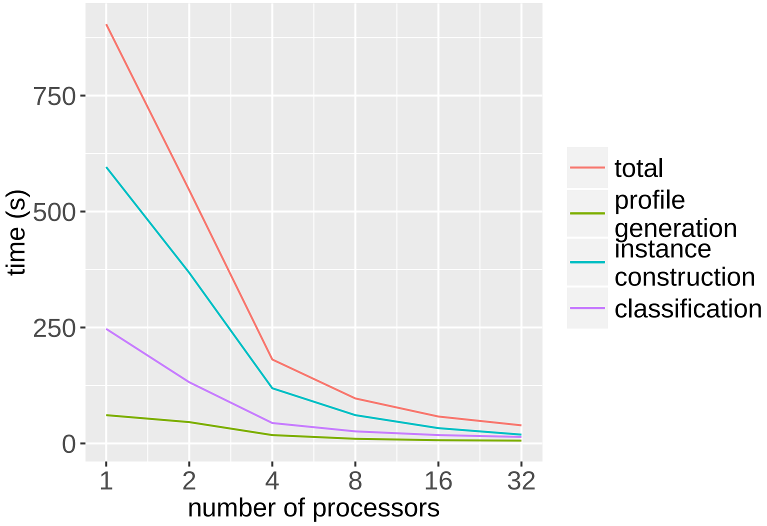

Supplement: S4 File — (PDF) [file pone.0161338.s004.pdf]
